# 4-(*o*-Tol­yl)piperazin-1-ium chloride

**DOI:** 10.1107/S1600536811044394

**Published:** 2011-10-29

**Authors:** Hoong-Kun Fun, Safra Izuani Jama Asik, B. Chandrakantha, Arun M. Isloor, Prakash Shetty

**Affiliations:** a X-ray Crystallography Unit, School of Physics, Universiti Sains Malaysia, 11800 USM, Penang, Malaysia; b Department of Chemistry, Manipal Institute of Technology, Manipal 576 104, India.; c Medicinal Chemistry Division, Department of Chemistry, National Institute of Technology-Karnataka, Surathkal, Mangalore, 575 025, India.; d Department of Printing, Manipal Institute of Technology, Manipal 576 104, India

## Abstract

In the title mol­ecular salt, C_11_H_17_N_2_^+^·Cl^−^, the piperazin-1-ium ring adopts a chair conformation with the aromatic ring in a pseudo-equatorial orientation. The dihedral angle between the benzene ring and the mean plane of the piperazin-1-ium ring is 51.22 (6)°. In the crystal, N—H⋯Cl hydrogen bonds link the mol­ecules into chains propagating in [100]. Weak C—H⋯π inter­actions also ocur.

## Related literature

For the medicinal applications of piperazine derivatives, see: Amir *et al.* (2004 ▶); Omar & AboulWafa (1986 ▶); El-Emam *et al.* (2004 ▶). For conformational analysis, see: Cremer & Pople (1975 ▶). For a related structure, see: Ben Gharbia *et al.* (2008 ▶).
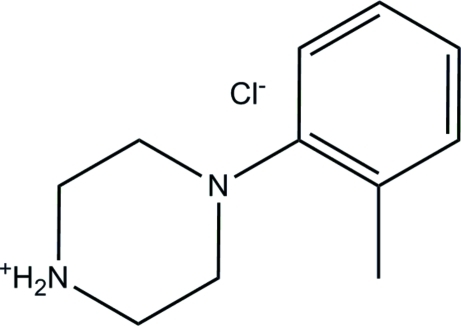


## Experimental

### 

#### Crystal data

C_11_H_17_N_2_^+^·Cl^−^
*M* *_r_* = 212.72
Orthorhombic, 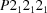

*a* = 8.1572 (2) Å
*b* = 11.2821 (3) Å
*c* = 12.4256 (3) Å
*V* = 1143.53 (5) Å^3^
*Z* = 4
Mo *K*α radiation
μ = 0.30 mm^−1^
*T* = 296 K
0.54 × 0.33 × 0.23 mm

#### Data collection

Bruker APEX DUO CCD diffractometer
Absorption correction: multi-scan (*SADABS*; Bruker, 2009) ▶ *T*_min_ = 0.854, *T*_max_ = 0.936
8246 measured reflections
4871 independent reflections
4172 reflections with *I* > 2σ(*I*)
*R*_int_ = 0.016

#### Refinement

*R*[*F* ^2^ > 2σ(*F* ^2^)] = 0.032
*wR*(*F* ^2^) = 0.092
*S* = 0.95
4871 reflections
128 parameters
H-atom parameters constrained
Δρ_max_ = 0.23 e Å^−3^
Δρ_min_ = −0.19 e Å^−3^
Absolute structure: Flack (1983 ▶), 1943 Friedel pairs
Flack parameter: 0.02 (4)

### 

Data collection: *APEX2* (Bruker, 2009) ▶; cell refinement: *SAINT* (Bruker, 2009) ▶; data reduction: *SAINT* ▶; program(s) used to solve structure: *SHELXTL* (Sheldrick, 2008 ▶); program(s) used to refine structure: *SHELXTL*; molecular graphics: *SHELXTL*; software used to prepare material for publication: *SHELXTL* and *PLATON* (Spek, 2009 ▶).

## Supplementary Material

Crystal structure: contains datablock(s) global, I. DOI: 10.1107/S1600536811044394/hb6474sup1.cif

Structure factors: contains datablock(s) I. DOI: 10.1107/S1600536811044394/hb6474Isup2.hkl

Supplementary material file. DOI: 10.1107/S1600536811044394/hb6474Isup3.cml

Additional supplementary materials:  crystallographic information; 3D view; checkCIF report

## Figures and Tables

**Table 1:** Hydrogen-bond geometry (Å, °) *Cg*2 is the centroid of C5–C10 ring.

*D*—H⋯*A*	*D*—H	H⋯*A*	*D*⋯*A*	*D*—H⋯*A*
N1—H1*N*1⋯Cl1^i^	0.87	2.26	3.1155 (10)	167
N1—H2*N*1⋯Cl1^ii^	0.87	2.23	3.0956 (10)	177
C3—H3*A*⋯*Cg*2^iii^	0.97	2.79	3.5342 (11)	134

Symmetry codes: (i) 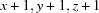; (ii) 
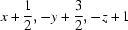
; (iii) 
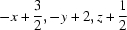
.

**Fig. 1.**
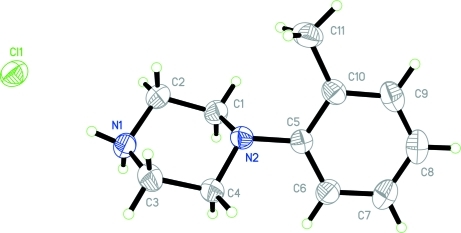
The structure of the title compound, showing 50% probability displacement ellipsoids.

**Fig. 2.**
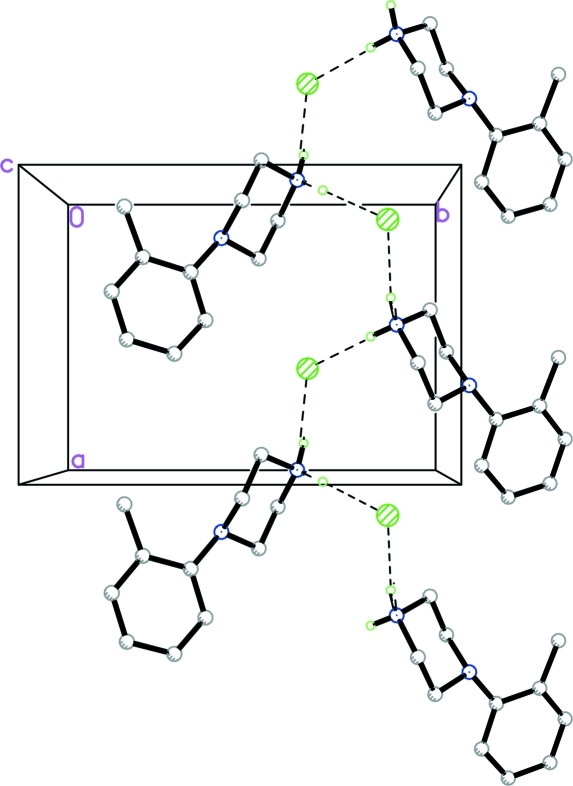
The crystal packing, viewed along the c axis, showing one-dimensional chains along the a axis. Hydrogen atoms that not involved in hydrogen bonding (dashed lines) are omitted for clarity.

## supplementary crystallographic information

### Comment

During the past years considerable evidence has been accumulated to demonstrate the efficacy of piperazine derivatives possessing antibacterial and antimicrobial activities (Amir *et al.*, 2004). For instance, Linezolid, Eperezolid, which are currently important antibiotics used for the treatment of microbial infections, contain the piperazine and morpholine ring in their structures (Omar & AboulWafa, 1986, El-Emam *et al.*, 2004).

As shown in Fig. 1, the asymmetric unit of the title compound contains a 4-(*o*-tolyl)piperazin-1-ium cation and a chloride anion. The benzene (C5–C10) ring and the mean plane of 4-(*o*-tolyl) piperazin-1-ium (C1–C4) make a dihedral angle of 51.22 (6)°. The piperazin-1-ium (N1/N2/C1–C4) adopts a chair conformation with puckering parameters Q = 0.5859 (11) Å, θ = 173.86 (11)° and φ = 3.0 (11)° (Cremer & Pople, 1975). It is also noted that the geometric parameters [d_a__v_(C–N) = 1.4653 (13) Å and d_a__v_ (C–C) = 1.5082 (16) Å for the 4-(*o*-tolyl)piperazin-1-ium moeity are in close agreement with those found in 4-(2,3-dimethylphenyl)piperazin-1-ium chloride monohydrate (Ben Gharbia *et al.*, 2008).

In the crystal (Fig. 2), N1—H1N1···Cl1 and N1—H2N1···Cl1 hydrogen bonds (Table 1) link the molecules into chains along the *a* axis. In addition, the crystal packing features weak intermolecular C—H···π interactions involving the benzene (C5–C10 ; centroid *Cg*2) ring with a distance of 3.5342 (11) Å.

### Experimental

To a stirred solution of 2-flurotoluene (2g, 0.0181 mol) and anhydrous potassium carbonate (3.7g, 0.027 mol) in dry acetonitrile (20 ml), piperizine-1-carboxylic acid tert butyl ester (3.38g, 0.0181 mol) was added dropwise at RT and reaction mixture was stirred at RT for 5h. After the completion of reaction, the reaction mixture was filtered and the filtrate was concentrated. The product (5g) was then dissolved with HCl in dioxane (25 ml) and stirred at RT for 2 h. The reaction mixture was concentrated through high vacuum. The crude product was recrystallised from hot ethanol to afford title compound as colourless blocks (3.0g, 66%).  M.p > 620K.

### Refinement

Atom H1N1 and H2N1 were located in a difference Fourier map and fixed to the positions with N–H = 0.8702 and 0.8662 Å. The remaining H atoms were positioned geometrically and refined using a riding modelwith C–H = 0.93–0.97 Å. The *U*_iso_ values were constrained to be 1.5*U*_eq_ of the carrier atom for methyl H atoms and 1.2*U*_eq_ for the remaining H atoms. A rotating group model was used for the methyl groups. 1943 Freidel pairs were used to determine the absolute configuration.

### Crystal data

**Table:**

C_11_H_17_N_2_^+^·Cl^−^	*F*(000) = 456
*M**_r_* = 212.72	*D*_x_ = 1.236 Mg m^−^^3^
Orthorhombic, *P*2_1_2_1_2_1_	Mo *K*α radiation, λ = 0.71073 Å
Hall symbol: P 2ac 2ab	Cell parameters from 3871 reflections
*a* = 8.1572 (2) Å	θ = 2.4–35.1°
*b* = 11.2821 (3) Å	µ = 0.30 mm^−^^1^
*c* = 12.4256 (3) Å	*T* = 296 K
*V* = 1143.53 (5) Å^3^	Block, colourless
*Z* = 4	0.54 × 0.33 × 0.23 mm

### Data collection

**Table:**

Bruker APEX DUO CCD diffractometer	4871 independent reflections
Radiation source: fine-focus sealed tube	4172 reflections with *I* > 2σ(*I*)
graphite	*R*_int_ = 0.016
φ and ω scans	θ_max_ = 35.5°, θ_min_ = 2.4°
Absorption correction: multi-scan (*SADABS*; Bruker, 2009)	*h* = −7→13
*T*_min_ = 0.854, *T*_max_ = 0.936	*k* = −10→18
8246 measured reflections	*l* = −6→20

### Refinement

**Table:**

Refinement on *F*^2^	Secondary atom site location: difference Fourier map
Least-squares matrix: full	Hydrogen site location: inferred from neighbouring sites
*R*[*F*^2^ > 2σ(*F*^2^)] = 0.032	H-atom parameters constrained
*wR*(*F*^2^) = 0.092	*w* = 1/[σ^2^(*F*_o_^2^) + (0.0554*P*)^2^ + 0.0493*P*] where *P* = (*F*_o_^2^ + 2*F*_c_^2^)/3
*S* = 0.95	(Δ/σ)_max_ < 0.001
4871 reflections	Δρ_max_ = 0.23 e Å^−^^3^
128 parameters	Δρ_min_ = −0.19 e Å^−^^3^
0 restraints	Absolute structure: Flack (1983), 1943 Friedel pairs
Primary atom site location: structure-invariant direct methods	Flack parameter: 0.02 (4)

### Special details

**Table:**

Geometry. All esds (except the esd in the dihedral angle between two l.s. planes) are estimated using the full covariance matrix. The cell esds are taken into account individually in the estimation of esds in distances, angles and torsion angles; correlations between esds in cell parameters are only used when they are defined by crystal symmetry. An approximate (isotropic) treatment of cell esds is used for estimating esds involving l.s. planes.
Refinement. Refinement of F^2^ against ALL reflections. The weighted R-factor wR and goodness of fit S are based on F^2^, conventional R-factors R are based on F, with F set to zero for negative F^2^. The threshold expression of F^2^ > 2sigma(F^2^) is used only for calculating R-factors(gt) etc. and is not relevant to the choice of reflections for refinement. R-factors based on F^2^ are statistically about twice as large as those based on F, and R- factors based on ALL data will be even larger.

### Fractional atomic coordinates and isotropic or equivalent isotropic displacement parameters (Å^2^)

**Table:**

	*x*	*y*	*z*	*U*_iso_*/*U*_eq_	
Cl1	0.37470 (3)	0.15197 (3)	0.10255 (2)	0.04298 (7)	
N1	1.02356 (12)	1.12619 (8)	1.00673 (8)	0.04027 (19)	
H1N1	1.1142	1.1422	1.0407	0.048*	
H2N1	0.9841	1.1900	0.9779	0.048*	
N2	0.81569 (10)	0.94027 (7)	0.93398 (6)	0.03177 (15)	
C1	0.93463 (14)	0.99338 (10)	0.86053 (8)	0.0381 (2)	
H1A	0.8839	1.0581	0.8214	0.046*	
H1B	0.9713	0.9347	0.8088	0.046*	
C2	1.07872 (14)	1.03893 (12)	0.92397 (10)	0.0461 (2)	
H2A	1.1337	0.9732	0.9590	0.055*	
H2B	1.1563	1.0765	0.8757	0.055*	
C3	0.89107 (15)	1.07704 (11)	1.07575 (8)	0.0427 (2)	
H3A	0.8502	1.1384	1.1234	0.051*	
H3B	0.9346	1.0134	1.1197	0.051*	
C4	0.75240 (13)	1.03055 (10)	1.00711 (8)	0.0380 (2)	
H4A	0.6679	0.9967	1.0526	0.046*	
H4B	0.7042	1.0949	0.9662	0.046*	
C5	0.69593 (12)	0.86761 (8)	0.88251 (6)	0.03052 (16)	
C6	0.53332 (14)	0.90410 (10)	0.87004 (8)	0.0382 (2)	
H6A	0.5015	0.9783	0.8951	0.046*	
C7	0.41906 (15)	0.83147 (12)	0.82098 (9)	0.0468 (3)	
H7A	0.3115	0.8571	0.8125	0.056*	
C8	0.46538 (18)	0.72070 (12)	0.78461 (10)	0.0507 (3)	
H8A	0.3895	0.6718	0.7507	0.061*	
C9	0.62468 (18)	0.68295 (10)	0.79889 (9)	0.0451 (2)	
H9A	0.6539	0.6076	0.7754	0.054*	
C10	0.74324 (14)	0.75387 (9)	0.84727 (7)	0.03504 (18)	
C11	0.91432 (17)	0.70764 (11)	0.86458 (11)	0.0482 (3)	
H11A	0.9117	0.6227	0.8691	0.072*	
H11B	0.9577	0.7396	0.9303	0.072*	
H11C	0.9827	0.7312	0.8054	0.072*	

### Atomic displacement parameters (Å^2^)

**Table:**

	*U*^11^	*U*^22^	*U*^33^	*U*^12^	*U*^13^	*U*^23^
Cl1	0.03237 (11)	0.04279 (13)	0.05376 (13)	−0.00052 (10)	0.00001 (10)	0.00279 (11)
N1	0.0329 (4)	0.0355 (4)	0.0524 (5)	−0.0008 (3)	−0.0083 (3)	−0.0036 (3)
N2	0.0313 (4)	0.0318 (4)	0.0322 (3)	−0.0009 (3)	0.0029 (3)	−0.0031 (3)
C1	0.0336 (5)	0.0428 (5)	0.0377 (4)	−0.0050 (4)	0.0063 (4)	−0.0029 (4)
C2	0.0295 (4)	0.0507 (6)	0.0580 (6)	−0.0033 (4)	0.0039 (4)	−0.0098 (5)
C3	0.0469 (6)	0.0424 (5)	0.0388 (4)	−0.0020 (5)	−0.0015 (4)	−0.0088 (4)
C4	0.0343 (4)	0.0393 (5)	0.0404 (4)	−0.0028 (4)	0.0055 (4)	−0.0101 (4)
C5	0.0328 (4)	0.0306 (4)	0.0282 (3)	−0.0023 (3)	0.0012 (3)	0.0013 (3)
C6	0.0340 (4)	0.0396 (5)	0.0410 (4)	−0.0005 (4)	−0.0011 (4)	0.0005 (4)
C7	0.0363 (5)	0.0571 (7)	0.0470 (5)	−0.0074 (5)	−0.0054 (4)	0.0010 (5)
C8	0.0522 (7)	0.0509 (7)	0.0489 (5)	−0.0197 (6)	−0.0047 (5)	−0.0018 (5)
C9	0.0574 (7)	0.0347 (5)	0.0433 (5)	−0.0106 (5)	0.0040 (5)	−0.0040 (4)
C10	0.0425 (5)	0.0295 (4)	0.0331 (4)	−0.0009 (4)	0.0021 (3)	0.0016 (3)
C11	0.0518 (7)	0.0381 (5)	0.0548 (6)	0.0117 (5)	−0.0019 (5)	−0.0026 (5)

### Geometric parameters (Å, °)

**Table:**

N1—C3	1.4869 (15)	C4—H4B	0.9700
N1—C2	1.4930 (15)	C5—C6	1.3974 (14)
N1—H1N1	0.8702	C5—C10	1.4098 (13)
N1—H2N1	0.8662	C6—C7	1.3827 (16)
N2—C5	1.4267 (12)	C6—H6A	0.9300
N2—C4	1.4593 (12)	C7—C8	1.382 (2)
N2—C1	1.4606 (13)	C7—H7A	0.9300
C1—C2	1.5056 (16)	C8—C9	1.379 (2)
C1—H1A	0.9700	C8—H8A	0.9300
C1—H1B	0.9700	C9—C10	1.3918 (16)
C2—H2A	0.9700	C9—H9A	0.9300
C2—H2B	0.9700	C10—C11	1.5053 (17)
C3—C4	1.5107 (15)	C11—H11A	0.9600
C3—H3A	0.9700	C11—H11B	0.9600
C3—H3B	0.9700	C11—H11C	0.9600
C4—H4A	0.9700		
			
C3—N1—C2	111.74 (9)	C3—C4—H4A	109.8
C3—N1—H1N1	114.6	N2—C4—H4B	109.8
C2—N1—H1N1	102.4	C3—C4—H4B	109.8
C3—N1—H2N1	106.2	H4A—C4—H4B	108.3
C2—N1—H2N1	112.0	C6—C5—C10	119.57 (9)
H1N1—N1—H2N1	110.1	C6—C5—N2	122.03 (9)
C5—N2—C4	115.97 (8)	C10—C5—N2	118.35 (9)
C5—N2—C1	114.24 (7)	C7—C6—C5	120.94 (11)
C4—N2—C1	109.74 (8)	C7—C6—H6A	119.5
N2—C1—C2	109.36 (9)	C5—C6—H6A	119.5
N2—C1—H1A	109.8	C6—C7—C8	119.73 (12)
C2—C1—H1A	109.8	C6—C7—H7A	120.1
N2—C1—H1B	109.8	C8—C7—H7A	120.1
C2—C1—H1B	109.8	C9—C8—C7	119.67 (11)
H1A—C1—H1B	108.3	C9—C8—H8A	120.2
N1—C2—C1	110.51 (9)	C7—C8—H8A	120.2
N1—C2—H2A	109.5	C8—C9—C10	122.20 (11)
C1—C2—H2A	109.5	C8—C9—H9A	118.9
N1—C2—H2B	109.5	C10—C9—H9A	118.9
C1—C2—H2B	109.5	C9—C10—C5	117.86 (11)
H2A—C2—H2B	108.1	C9—C10—C11	120.45 (10)
N1—C3—C4	110.37 (8)	C5—C10—C11	121.65 (10)
N1—C3—H3A	109.6	C10—C11—H11A	109.5
C4—C3—H3A	109.6	C10—C11—H11B	109.5
N1—C3—H3B	109.6	H11A—C11—H11B	109.5
C4—C3—H3B	109.6	C10—C11—H11C	109.5
H3A—C3—H3B	108.1	H11A—C11—H11C	109.5
N2—C4—C3	109.21 (9)	H11B—C11—H11C	109.5
N2—C4—H4A	109.8		
			
C5—N2—C1—C2	−164.57 (9)	C10—C5—C6—C7	−1.76 (15)
C4—N2—C1—C2	63.23 (11)	N2—C5—C6—C7	−179.23 (10)
C3—N1—C2—C1	53.08 (13)	C5—C6—C7—C8	0.65 (17)
N2—C1—C2—N1	−57.39 (13)	C6—C7—C8—C9	0.87 (18)
C2—N1—C3—C4	−53.24 (12)	C7—C8—C9—C10	−1.31 (18)
C5—N2—C4—C3	165.32 (8)	C8—C9—C10—C5	0.20 (16)
C1—N2—C4—C3	−63.38 (11)	C8—C9—C10—C11	177.96 (11)
N1—C3—C4—N2	57.89 (12)	C6—C5—C10—C9	1.32 (13)
C4—N2—C5—C6	22.41 (12)	N2—C5—C10—C9	178.88 (9)
C1—N2—C5—C6	−106.74 (10)	C6—C5—C10—C11	−176.42 (10)
C4—N2—C5—C10	−155.10 (9)	N2—C5—C10—C11	1.14 (13)
C1—N2—C5—C10	75.76 (11)		

### Hydrogen-bond geometry (Å, °)

**Table:**

Cg2 is the centroid of C5–C10 ring.

**Table:**

*D*—H···*A*	*D*—H	H···*A*	*D*···*A*	*D*—H···*A*
N1—H1N1···Cl1^i^	0.87	2.26	3.1155 (10)	167
N1—H2N1···Cl1^ii^	0.87	2.23	3.0956 (10)	177
C3—H3A···Cg2^iii^	0.97	2.79	3.5342 (11)	134

Symmetry codes: (i) *x*+1, *y*+1, *z*+1; (ii) *x*+1/2, −*y*+3/2, −*z*+1; (iii) −*x*+3/2, −*y*+2, *z*+1/2.
